# How can we facilitate research on the risks and potential benefits of novel psychoactive substances?

**DOI:** 10.1016/j.eclinm.2026.104030

**Published:** 2026-06-19

**Authors:** Ramaekers JG, Bade R, Hall W

**Affiliations:** a Faculty of Psychology and Neuroscience, Maastricht University, Maastricht, the Netherlands; b Queensland Alliance for Environmental Health Sciences, The University of Queensland, Australia; c National Centre for Youth Substance Use Research, The University of Queensland, St Lucia, Australia

**Keywords:** Novel psychoactive substances, Drug policy, Clinical research, Harms, Benefits

## Abstract

Novel psychoactive substances (NPS) are synthetic compounds designed to mimic illicit drugs while circumventing international drug regulations. Commonly marketed as “research chemicals” or “legal highs”, these substances remain poorly understood, with limited evidence regarding their pharmacology, risks, and therapeutic potential. Current drug policies often place NPS in the strictest legal schedules, restricting the clinical and pharmacological research needed to assess both harms and possible medical benefits. This paper advocates for a public health-oriented regulatory framework that balances harm reduction with controlled scientific access. Existing clinical evidence indicates that some NPS present significant risks, whereas others may possess therapeutic value. To address this complexity, an evidence-based four-stage scheduling framework is proposed: Early Surveillance to identify emerging substances and harms; Provisional Scheduling to permit access to regulated research; Controlled Research to investigate pharmacology, safety, subjective and neurocognitive effects, abuse liability, and therapeutic potential; and Reclassification based on integrated clinical, toxicological, and public health evidence. This approach would reduce research barriers and support more adaptive, evidence-based regulation and more balanced drug policies.

**Funding:**

None.

## Introduction

Novel psychoactive substances (NPS) are mind-altering compounds that fall outside the control of the 1961 and 1971 UN drug treaties and the 1988 convention against illicit traffic. These UN schedules classify drugs based on evaluations by WHO of their abuse potential, health risks, and medical value, with stricter schedules imposing tighter international controls. NPS are designed to bypass drug laws and sold as substitutes for illicit drugs, such as amphetamines, heroin, cocaine, and cannabis under misleading labels, such as “research chemicals” and “legal highs”. NPS comprise a relevant segment of the global drug landscape, with over 1400 different NPS detected internationally since 2009.^1^^,^^2^

Scientific understanding of their pharmacological profiles, risks, and possible therapeutic applications is limited. National drug classification systems often place NPS in the most restrictive schedules, indicating high harm potential and no accepted medical use, frequently based on UN conventions and, in some cases, without conducting any formal risk evaluation at all. However, such policies may constrain the research necessary to evaluate both their risks and potential benefits. The latter includes investigations into potential therapeutic applications of NPS and the mechanistic basis of their effects, while the former includes toxicological monitoring of possible cardiovascular, behavioural and psychiatric adverse events, as well as addiction liability.

A public health-oriented regulatory system would shift from reactive prohibition to allowing controlled access for rigorous clinical and pharmacological research on their harms and benefits. Facilitating research does not have to imply broader public availability or reduced risk messaging, as the substance can remain tightly controlled for the public. This would also be in line with UN drug conventions that do permit the use of controlled substances for therapeutic and research purposes and primarily aim to prohibit their possession and use in non-medical contexts.^3^^,^^4^

However, few clinical trials on NPS have been conducted. This reflects the challenges that academic and medical institutions face in securing licences to handle scheduled substances, the reluctance of Institutional Review Boards to approve first-in-human studies without extensive preclinical safety data, the complex regulatory processes for importing and exporting these compounds, the limited availability of high-purity formulations suitable for clinical use, and the high costs associated with meeting good manufacturing practice standards.^5^

Limited trials of NPS have nevertheless demonstrated significant value. They have produced controlled safety data that meaningfully complements retrospective observations of naturalistic use. Controlled clinical trials have enabled systematic evaluation of both risks and benefits, showing that some NPS are more likely to cause harm, while others may have potential therapeutic benefits.

## Existing evidence

Synthetic cannabinoids are an NPS class associated with disproportionate harm. Unlike plant-derived cannabis, which contains partial agonists at CB1 receptors, many synthetic cannabinoids are ultrapotent, full CB1 agonists. They have receptor binding affinities up to 100-fold greater than Δ^9^-tetrahydrocannabinol (Δ^9^-THC), the primary psychoactive component of cannabis.^6^ As a result, they have a much narrower safety margin and in case reports and emergency department admissions they have been linked to severe neurological and cardiovascular toxicity, including seizures, loss of consciousness, tachycardia, and hypertension.^6^^,^^7^

Controlled clinical studies with JWH-018, an early representative of this class, have provided mechanistic insight into the toxicity of synthetic cannabinoids. Even small to moderate doses in otherwise healthy individuals have produced marked impairment and intense psychotomimetic effects, including confusion, amnesia, derealisation, and depersonalisation.^8^, ^9^, ^10^ These effects were substantially more pronounced than those observed with cannabis at psychotropic dose equivalence.^10^ Additionally, the high inter-individual variability in delivery efficiency and serum concentrations resulted in unpredictable dosing and increased overdose risk.^11^ These controlled studies highlight the disproportionate harm posed by synthetic cannabinoids and underscore the need for targeted public health guidance and harm-reduction strategies.

Psychedelic drugs and 3,4-methylenedioxymethamphetamine (MDMA) show that strict drug scheduling may inhibit clinical research on therapeutic applications. Classical psychedelics such as psilocybin, lysergic acid diethylamide, 5-methoxy-N,N-dimethyltryptamine, and N,N-dimethyltryptamine are now under investigation for the treatment of depression and anxiety,^12^^,^^13^ after decades during which research was constrained by regulatory restrictions. MDMA-assisted psychotherapy has demonstrated efficacy in post-traumatic stress disorder (PTSD),^14^ although obtaining regulatory approval has remained challenging. Together, these cases illustrate how restrictive scheduling frameworks can foreclose scientific discovery, a dynamic that may similarly hinder clinical investigation into NPS.

A range of structurally modified, novel psychedelics, including substituted phenethylamines, tryptamines, and lysergamides, are used in recreational settings to induce psychedelic or entactogenic states.^15^^,^^16^ These drugs may possess therapeutic potential. Among these, only 2C–B has been examined under controlled clinical conditions. It has been found to produce a shorter and comparatively “lighter” psychedelic experience than psilocybin, potentially making it more tolerable and easier to use in clinical settings.^17^ Likewise, biotech companies that have recognised the value of functional modifications are actively engaged in developing next generation psychedelics with greater tolerability, efficacy and feasibility of clinical use.^18^

Synthetic cathinones are another major class of NPS whose therapeutic potential has been underexplored. These are structural analogues of cathinone, a naturally occurring psychostimulant found in the leaves of the khat plant. Synthetic cathinones act as substrates for monoamine transporters that stimulate to varying degrees the release and inhibit the reuptake of monoamines. In a small number of controlled and observational human studies, synthetic cathinones such as mephedrone, methylone, alpha-PVP and 3-methylmethcathinone produced cardiovascular, psychostimulant and psychotomimetic effects that are similar to amphetamine.^19^, ^20^, ^21^, ^22^

Most synthetic cathinones have been classified internationally as controlled substances with no recognised medical use based on reports of misuse and severe intoxication when taken in high doses or with alcohol and other drugs. Yet, in the mid-20th century, cathinone derivatives were explored for therapeutic use in appetite suppression, the treatment of depression, and the management of pain.^23^^,^^24^ Today, both established and emerging therapeutic applications are recognised. Bupropion, the N-tert-butyl analogue of cathinone, is an approved medication for depression and smoking cessation. Methylone has received Breakthrough Therapy designation from the US Food and Drug Administration (FDA) for the treatment of PTSD. A placebo-controlled phase 2 trial demonstrated that 4 once-weekly oral dosing sessions of methylone (150 mg followed by 100 mg or placebo), without psychotherapy, induced a clinical relevant reduction in symptoms of PTSD that lasted for at least 64 days.^25^ Low to moderate doses of 3-methylmethcathinone have produced prolonged analgesic effects in healthy volunteers, with only benign side effects.^24^ Further systematic clinical research could clarify whether selected compounds in this class can be safely^26^ developed as treatments for pain, mood disorders, or other conditions.

## The policy challenge

The challenge for policy makers is how to regulate NPS in ways that protect public health without blocking research on their risks and benefits. Fortunately, most NPS do not require legislative action or extensive study, because many disappear quickly from the market. Wastewater surveillance is a promising way to monitor the emergence, persistence and disappearance of NPS.^27^ Broader drug intelligence and early warning system approaches, including emergency department, coronial, police seizure, drug checking services data are also critical for early detection and risk communication.^28^ These can also potentially identify NPS that are frequently used in the absence of a high prevalence of adverse events.

Only when a particular compound persists and is judged to pose a perceived health risk, is it generally brought under international control. Approximately 87 NPS have been scheduled internationally since 2014,^1^ including the substances discussed above. Scheduled NPS that persist on the market, regardless of the market dynamics underlying their persistence, constitute important targets for exploratory research that potentially would hold relevance to both clinical and recreational use.

These substances are typically placed into the most restrictive schedule of harmful drugs, in the absence of comprehensive data on their pharmacology, toxicity, or therapeutic potential. A more balanced approach would place newly controlled NPS in a lower, less restrictive schedule that allowed regulated clinical research to evaluate both their harms and benefits. If subsequent studies show that a substance's risks clearly outweigh its potential value, it could be moved to a more restrictive schedule.

Drug classification operates like a regulatory ratchet because it is far easier to increase than reduce restrictions. This is because a higher standard of evidence is required for down-scheduling than that required for the initial classification and because the restrictions arising from the classification prevent the research required to justify any down-scheduling. This is especially true if the substance falls under Schedule I (and the even more restrictive sub-schedule IV) of the 1961 UN Convention or under Schedule 1 of the 1971 convention because broad international agreement is required for any classificatory amendments. For example, in 2020, the UN Commission on Narcotic Drugs voted to remove cannabis and cannabis resin from Schedule IV of the 1961 UN Convention after 59 years and decades of broad international debate, following recommendations from WHO and its Expert Committee on Drug Dependence. The vote passed narrowly with 27 countries in favour, 25 against, and one abstention. Cannabis remained classified under Schedule I due to its potential for abuse, but was no longer being regarded among the most harmful substances lacking significant therapeutic or medical value. The decision was intended to support scientific research and could encourage countries to expand or legalise medical cannabis programs.^29^

An alternative approach would avoid making drug controls politically or legally resistant to revision when new evidence demonstrates medical benefits or lower levels of harm, and place emerging substances into a designated schedule that permitted controlled medical use under licenced supervision, while preventing recreational trafficking. Because the therapeutic potential of NPS is often unknown at the time of scheduling, placing them in a less restrictive schedule could enable the research needed to assess their medical benefits and harms.

## A framework for the evidence-based scheduling and evaluation of NPS

**Fig. 1:**
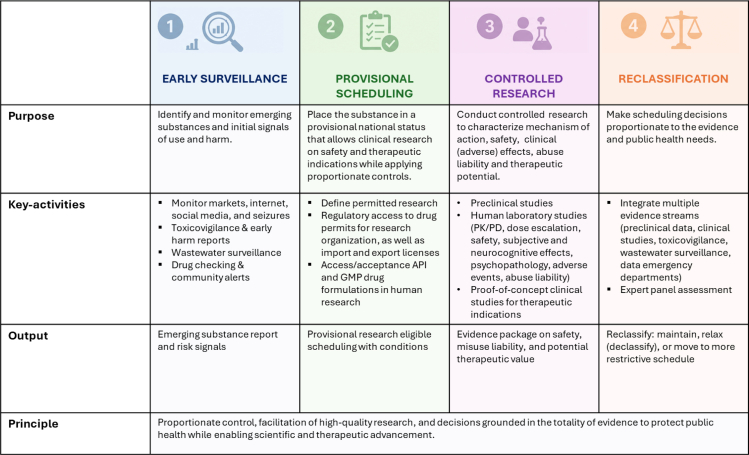
A dynamical, four-staged practical framework for evidence-based scheduling and evaluation of risks and benefits of NPS.

An illustrative, four-stage framework for the evidence-based scheduling and evaluation of NPS is provided in Fig. 1. The framework begins with Early Surveillance, which focuses on detecting emerging substances and early signals of harm through toxicovigilance, online monitoring, wastewater surveillance, and drug-checking initiatives. This stage would generate an initial risk signal and emerging substance report. The second stage, Provisional Scheduling, introduces a temporary regulatory status that enables controlled research under proportionate safeguards, allowing licenced organisations to conduct approved studies through regulated access pathways, including research permits, import/export licences, and access to active pharmaceutical ingredient or good manufacturing practices drug formulations. The third stage, Controlled Research, involves preclinical and human laboratory investigations assessing pharmacology, safety, adverse effects, abuse liability, psychopathology, neurocognitive outcomes, and therapeutic potential. It would produce an evidence package evaluating both risks and possible clinical value. The final stage, Reclassification, would integrate evidence from multiple complementary sources, including human laboratory studies, observational toxicovigilance, wastewater surveillance, drug-checking data, and emergency department presentations. This stage would support expert review and scheduling decisions, whereby substances may remain in the current schedule, be moved to a less restrictive category (including declassification) or be placed under more restrictive control. Overall, the framework would promote dynamic and proportionate regulation, iterative evidence review, and evidence-based scheduling decisions for NPS.

The Act on Psychomodulatory Substances introduced in the Czech Republic in 2025 provides a practical example of flexible and dynamic drug scheduling designed to regulate emerging psychoactive substances more adaptively than traditional drug laws. The system establishes three categories: addictive substances (strictly prohibited narcotics or psychotropics, highly controlled medical use), scheduled psychoactive substances (new or uncertain substances under temporary restriction requiring scientific research), and psychomodulatory substances (lower-risk substances allowed to be marketed for human consumption in a non-medical context under strict regulation). Authorities can rapidly place new substances into the scheduled psychoactive substances category pending scientific assessment. Based on evidence of harm, substances may later be reclassified either as regulated psychomodulatory substances or as fully controlled narcotics. Since these changes can be made through administrative regulations rather than new parliamentary legislation, the system allows much faster adaptation to scientific and market developments.^30^ For example, hexahydrocannabinol (HHC) and related synthetic cannabinoids were rapidly moved into restrictive categories after poisonings among minors,^31^ while kratom (mitragyna speciosa) and low-THC cannabis products (up to 1% THC) were ultimately placed into the psychomodulatory category^32^ rather than being banned. However, following a notification procedure at the European Commission, which argued that cannabis is an internationally controlled narcotic regardless of its origin or THC content, low-potency cannabis was removed from the list of psychomodulatory substances, which again confirms the restrictive nature of the global drug control system. Nevertheless, this model is innovative because it creates a dynamic legal pathway between full prohibition and full legalisation.

The aim of dynamic drug scheduling is to promote continuous, iterative scientific research to support evidence-based drug classification. Governments and funding agencies should support systematic assessments of the potential harms and potential benefits of NPS in order to enable more evidence-informed regulation. For example, scientific research became central to debates over the scheduling of kratom under the US Controlled Substances Act after the Drug Enforcement Administration announced plans in 2016 to temporarily place its primary alkaloids in Schedule I. Kratom is a tropical Southeast Asian plant traditionally used for analgesia, mood enhancement, and the relief of physical fatigue. The proposal was withdrawn after scientific criticism, public comments, and calls for further evaluation, pending the collection of additional evidence on kratom's risks and potential benefits.^33^

US federal agencies, including the FDA and the National Institutes of Health, have acknowledged the lack of controlled clinical trials on botanical kratom and concentrated extracts containing 7-OH mitragynine. In many countries these are marketed as “legal highs” in the form of tablets, herbs, gummies or mixed beverages. In response, federal research funding has been directed toward human clinical studies to evaluate the safety, pharmacology, and dependence liability of kratom-derived products given their notable activity at the μ-opioid receptor. These regulatory agencies have encouraged academic researchers and industry to investigate whether kratom or its alkaloids may have therapeutic value in pain management or in mitigating opioid withdrawal symptoms. These efforts aim to provide sound public health evidence to determine whether federal regulation is warranted.

If comparable evidence-based strategies were implemented globally and extended to other major NPS, many current obstacles to research could be reduced, enabling more responsible, innovative, and well-regulated investigations into their safety and therapeutic potential. Such strategies could also help inform recreational NPS users who, irrespective of scheduling, use these substances for non-medical purposes perceived as meaningful, including mood enhancement, sociability, pleasure, creativity, or recreation, often despite limited knowledge of potential harms. Current drug control systems rarely acknowledge such non-medical use because regulatory legitimacy is typically tied to medical utility and safety. This creates an inherent tension, as the psychoactive effects sought by users often overlap with the intoxicating properties regulators seek to control. Acknowledging that psychoactive substances are used in both medical and non-medical contexts reinforces the need for robust scientific research into their harms and benefits as the foundation for adaptive evidence-based regulation and more balanced drug policies.

## Contributors

JR wrote the manuscript. RB and WG reviewed and edited the manuscript.

## Declaration of interests

WH declares receipt of payment for expert testimony on the psychosocial effects of parents supplying cannabis to their early adolescent children. JR and RB declare no competing interests.
